# Parent perceptions of the quality of life of pet dogs living with neuro-typically developing and neuro-atypically developing children: An exploratory study

**DOI:** 10.1371/journal.pone.0185300

**Published:** 2017-09-27

**Authors:** Sophie S. Hall, Hannah F. Wright, Daniel S. Mills

**Affiliations:** School of Life Sciences, University of Lincoln, Brayford Pool, Lincoln, United Kingdom; University of North Carolina at Chapel Hill, UNITED STATES

## Abstract

There is growing scientific and societal recognition of the role that pet dogs can play in healthy development of children; both those who are neuro-typically developing and those who live with a neuro-developmental disorder, such as autism or attention deficit hyperactivity disorder. However, little attention has been paid to how living with children positively and negatively affects quality of life of a pet dog. In this exploratory study we conducted semi-structured interviews with parents of neuro-typically developing children (n = 18) and those with a neuro-developmental disorder (n = 18) who owned a pet dog, until no new factors were identified. Living with children brought potentially positive benefits to the dog’s life including: imposition of a routine, participation in recreational activities and the development of a strong bond between the child and the dog. The importance of maintaining a routine was particularly prevalent in families with children with neuro-developmental disorders. Potential negative factors included having to cope with child meltdowns and tantrums, over stimulation from child visitors, harsh contact and rough and tumble play with the child. The regularity and intensity of meltdowns and tantrums was particularly evident in responses from parents with children with a neuro-developmental disorder. However, child visitors and rough play and contact were mentioned similarly across the groups. Protective factors included having a safe haven for the dog to escape to, parent’s awareness of stress signs and child education in dog-interaction. Parents were also asked to complete a stress response scale to provide an initial quantitative comparison of stress responses between dogs living with the two family-types. Parents with neuro-typically developing children more frequently observed their dog rapidly running away from a situation and less frequently observed their dog widening their eyes, than parents with children with a neuro-developmental disorder. We propose the development of a stress audit based on the findings reported here, to prevent potential dangerous situations, which may lead to dog bites and dog relinquishment and allow owners to maximise the benefits of dog ownership.

## Introduction

It is estimated that over 8.5 million dogs in the UK are living as family pets [1], yet there is little research considering the stressors these dogs encounter [2–3]. Despite growing awareness of the important relationship between stress, dog quality of life and problematic behaviour, research in this area is still in its infancy [4]. While some studies have sought to identify and evaluate dogs’ stress responses to specific stimuli such as experimental or training stressors like the delivery of electric shocks [5–7], there appears to be a lack of information about when dogs might show general signs of stress in the home environment. Although the presence of younger children and teenagers in the home are a potential risk factor for dog bites [8–10], it is not clear that such bites are necessarily closely related to particularly stressful circumstances for the dog [11,12].

Spatial restrictions [13, 14] and noise disturbance [15,16] can cause stress and negatively impact on the quality of life of dogs. Family composition and dynamic may influence the extent to which these and other factors are important in a given home, but this subject seems to be relatively unexplored. The majority of dog owners believe they care for their dogs’ needs and have a sound understanding of their dogs’ emotional well-being, but evidence suggests that they may not be very sensitive to the behavioural signs of low level stress or arousal [17, 18], even though behavioural signs relating to stress and anxiety are those that often viewed as most problematic by their owners [19]. Chronic “low grade” stressors such as social tension, as opposed to specific stressful events, have been shown in other species to have a significant impact on health and thus quality of life [20] and a similar impact on dogs has been suggested [4, 21]. Nonetheless, little research has explored how family factors can affect stress and general quality of life of dogs. This is of particular importance due to human as well as animal welfare concerns. These concerns range from the risk of a potentially catastrophic event, such as a serious dog bite, to the potential to maximise the benefits of dog ownership on human health. There is a growing literature indicating positive effects of pet ownership on family life [22–36], including it’s potential to promote child development to some degree [37]. However, these benefits are clearly not an inevitable consequence of acquiring a dog, and a stressed dog may seriously limit the potential benefits of ownership.

The stress and anxiety reducing benefits may be of particular value in families, both those with neuro-typically developing children [28–31] and those with neuro-atypically developing children, such as those with autism spectrum disorder (ASD) [32–34], where there may be particular benefit to the primary carer of the child [35–36]. ASD and attention deficit hyperactivity disorder (ADHD) are two of the most common neuro-developmental disorders observed in childhood [38]. Briefly, ASD is characterised by difficulties in verbal and non-verbal communication and ADHD by problems with attending and inhibiting behaviour [39], these difficulties cause significant impairment and a reduced ability to function.

Only one paper is known to the authors which has investigated potential stressors for dogs living with children with autism [40]. This study interviewed 11 participants, all of whom had acquired a trained autism assistance dog, as opposed to a pet dog. Given that autism assistance dogs undergo specific training prior to being placed with children, it is possible that non-prepared pet dogs experience, and respond to, different types of stressors and it is not known in what way this might differ for dogs living in families with a neuro-typically developing child. This exploratory study aimed to identify parent perspectives of the issues which positively and negatively affect the quality of life of pet dogs living with children who are neuro-typically developing and children who have a neuro-developmental disorder (ASD and/or ADHD). To this affect we primarily used a qualitative research approach involving interviewing to redundancy.

## Methods

The research process was approved by the University of Lincoln’s, College of Science ethics committee and abided by the British Psychological Society Ethics Code of Conduct (2009). Fully informed, written consent was obtained from all participants prior to the interviews (Ethical approval ID: CoSREC109).

### Participants

Participants were recruited on a voluntary basis from national targeted press advertisements, contacts with family support groups, schools and charitable dog organisations. Participants were sent a study information sheet and consent form upon initial contact with the study lead (SH). All participants who met the eligibility criteria (owned a family dog, for at least 2 years, and had a child aged 4–10 years) were included in the study.

Thirty-six parents who met the eligibility criteria were recruited. Demographic data relating to the child, family (Table 1) and dog (Table 2) were collected (in each group 17 parents returned the completed demographic forms). Eighteen parents (2 male) had a child with a clinically confirmed neurodevelopmental disorder of ASD and/or ADHD; these formed the neuro-developmental disorder (NDD) group. Parents were required to send an anonymised copy of their child’s diagnosis to the researchers, all children had received a primary diagnosis of ASD or ADHD through Child Adolescent Mental Health Services (CAMHS) or community paediatrics. Because of the heterogeneous nature of ASD and ADHD we did not include exclusion criteria relating to the condition for participation in the study, in order to obtain a sample that reflected the disparity of characteristics of families in the general population.

**Table 1:** Demographic information for the participant groups detailing child and family information.

	Neuro Typically Developing*	Neuro Developmental Disorder*
**Primary Neuro Developmental Diagnosis**		
No diagnosis	100%	0%
Autism Spectrum Disorder (ASD)	0%	72%
Attention Deficient Hyperactivity Disorder (ADHD)	0%	28%
Strengths & Difficulties Scores** **(**Average ± Standard Deviation)		
Total difficulties	6.64 ± 4.01	24.35 ± 7.16
Emotional problems	1.29 ± 1.44	5.29 ± 1.99
Conduct problems	1.17 ± 1.59	4.64 ± 3.04
Hyperactivity score	3.23 ± 2.19	8.00 ± 2.73
Peer problems score	0.94 ± 1.08	6.41 ± 2.15
Prosocial score	7.76 ± 2.65	4.76 ± 2.51
**Child’s Age**		
Average ± Standard Deviation	6.5 years ± 2.5	7.8 years ± 1.8
**Child’s Gender**		
Male	53%	82%
Female	47%	18%
**Other Children in the Household**		
Average ± Standard Deviation	2.5 ± 1.4	2.4 ± 1.4
**Carer Relationship**		
Mother main carer to child	100%	89%
Father main carer to child	0%	5.5%
Grandparent main carer to child	0%	0%
Foster / Adopted child	0%	5.5%
Mother main carer to dog	94%	94%
Father main carer to dog	6%	0%
Child main carer to dog	0%	6%
**Carers in the Household**		
No carers	100%	94%
Family carers	0%	6%
Formal carers	0%	0%

*Completed data from 17 participants

**Scoring Bands: Total difficulties (Close to average: <13; Slightly raised/lowered: 14–16; High/low: 17–19; Very high/low:>20). Emotional problems (Close to average: <3; Slightly raised/lowered: 4; High/low: 5–6; Very high/low:>7). Conduct problems (Close to average: <2; Slightly raised/lowered: 3; High/low: 4–5; Very high/low:>6). Hyperactivity score (Close to average: <2; Slightly raised/lowered: 3; High/low: 4; Very high/low:>5). Peer problems score (Close to average: <2; Slightly raised/lowered: 3; High/low: 4; Very high/low:>5). Pro-social score (Close to average: 8–10; Slightly raised/lowered: 3; High/low: 6; Very high/low: 0 = 5).

**Table 2:** Demographic information for the participant groups detailing dog information.

	Neuro Typically Developing*	Neuro Developmental Disorder*
**Age**		
Average ± Standard Deviation	5.7 years ± 2.8	4.1 years ± 2.5
**Breed Type**		
Small Breed	29.4%	18.8%
Large Breed	41.1%	37.5%
Cross Breed	29.4%	43.8%
**Sex/ neuter status**		
Male entire	6%	13%
Male neutered	35%	56%
Female entire	29%	19%
Female neutered	29%	13%
**Length of Time Owned**		
Mean ± Standard Deviation	5.1 years ± 2.3	3.4 years ± 2.4
**Dog Source**		
Breeder	47%	31%
Private Family	24%	38%
Rescue	29%	31%
**Dog Training**		
No formal training	47%	44%
Assistance pet dog workshops	0%	25%
Puppy socialisation	47%	25%
Obedience training	6%	6%
**Number of Dogs in the Household**		
1 dog	59%	75%
2 dogs	35%	18%
3 dogs	6%	6%
**Sources of Support for Owners on Dog Ownership Advice** (can select multiple responses)		
Internet	4	3
Friends / Family	9	0
Vets	12	8
Support dog organisations	2	4
Behaviourist	6	7

*Completed data from 17 participants

Eighteen parents (all female) had a child with no clinically confirmed, or suspected, neuro-developmental disorder; these formed the neuro-typically developing (NTD) group. In families where more than one child met the age criteria (n = 7), parents were asked to select the child who they believed had the closest relationship with the dog. In families where more than one dog was owned parents (see Table 2) were asked to respond with regards to the dog that they believed had the closest relationship with their child in question.

### Child strengths and difficulties

Parents were asked to complete the Strength and Difficulties Questionnaire (SDQ) [41, 42] to provide confirmation of their child’s difficulties. The forms were scored in line with the scoring manual. The SDQ is a brief behavioural screening questionnaire comprised of 25 items and 5 scales (emotional symptoms, conduct problems, hyperactivity/inattention, peer-relationship problems and pro-social behaviour). The respondents answered questions on a three-point scale (not true, somewhat true, certainly true) and five questions were reverse scored.

### Dog stress response checklist

The stress response checklist comprised a list of 22 behaviours identified as indicators of increased stress and arousal in dogs (nose licking, blinking, turning away, staring, gaze aversion, panting, whining, barking, growling, snapping, biting, lip-licking, tense mouth, wide eyes, shaking, cowering, hiding, rapid running away, slow running away, walking away, restlessness, and repetitive behaviours such as tail chasing and constant licking). The behaviours were selected from a brief review of the literature [4, 43–45] and evaluated for suitability and face validity by a specialist in veterinary behavioural medicine (DM), before being reviewed by the project advisory team for readability and comprehensiveness. The project advisory group was made up of five members, including professionals in the field of neuro-developmental disorders, veterinary-behaviour, assistance dog work, academics and parents who own a family pet dog. Participants were asked to mark when they had seen the dog show these behaviours across eight categories of possible interactions between the child and dog. The categories were selected based on consultations within our project team and advisory group and included: ***Resting***: Sitting, laying, sleeping with my child. ***Playing***: Playing with my child. ***Walking***: Walking with my child on or off the lead. ***Dog eats***: Dog is eating near my child. ***Child eats***: Child is eating near my dog. ***Physical***: Being stroked, cuddled, groomed by my child. ***Car***: In the car with my child. ***Running***: Running or chasing with my child. Parents were instructed to record which behaviours they had seen their dog display in the past month during these child-dog interactions, therefore scores represent the presence/absence of observations of the specific behaviours as opposed to frequency counts. Parents were informed to select more than one behaviour per category of interaction if relevant. Participants did not receive any training on recognising these behaviours in order to gain an insight into parent’s everyday observations of their dogs that they naturally attend to.

### Interview item generation and analysis

The interview questions were initially compiled following a review of the existing literature [35, 40, 46–47] and then circulated to the project advisory group for additional input and comments. The resulting interview schedule addressed specific areas associated with dog ownership in families with children. These areas included (i) Background: dog’s personality, dog’s health, and home environment; (ii) Routine: normal routine, how the child affects this routine and how the dog responds to changes in routine; (iii) Relationship between the child and dog: typical interactions between the child and dog, behaviours displayed by the dog when with the child, whether the dog seeks out anyone else when interacting with child; (iv) Communications between the child and dog: interaction between the dog and the child that the dog appears to enjoy the most and like the least. The interviewer adopted a friendly approach during the interview, but avoided providing feedback or personal discussion on any of the points made by the parents during the interview to avoid biasing the process.

Audio recordings were anonymised during transcription, with reference to names removed. The transcripts were not returned to participants, on nine occasions words were identified as missing from the transcripts, these were resolved by the researchers listening to the recordings to identify the missing word. Initial analysis was conducted by SH, following the guidelines of Braun and Clarke [48]. Each coding unit was assigned exclusively to one rather than multiple categories to create well defined coding categories [49] which decribed the data. To ensure validity and reliability of data interpretation, the transcripts were second coded by another researcher (HW). Themes emerged from the data and were largely consistent between coders, where contradictory coding was apparent, the coders resolved this through discussion until consensus was reached. We sought to understand factors affecting the quality of life of dogs through the words of the participants, as opposed to the researcher’s expectations. Nonetheless, we recognise that it is difficult to be truly objective when interpreting qualitative data and therefore our background and expertise will have in some way influenced the defining of themes.

### Procedure

Data collection took part between March and June 2016. Prior to the interviews commencing, all participants had read an information sheet and signed a consent form. In the week before the interview date participants were requested to complete the demographic questionnaire, the SDQ and the Dog Stress Response Checklist. A few parents requested that the questionnaires and checklist be sent in the post. Not all parents returned all of the forms complete (completion numbers for each form are presented with their respective data). All interviews were conducted by SH and audio-recorded to aid subsequent transcription. Interviews took place individually with each participant over the telephone (Average duration: 21.01 minutes: Range: 11.04–34.04 minutes). The interviews were semi-structured and guided by the interview schedule. By utilising a semi-structured approach, the researcher was able to ask additional questions based on the interviewees’ responses. An interview-to-redundancy technique was used to determine the final sample size (recruitment continued until no additional qualitatively different responses could be identified from the ongoing interviews, to ensure redundancy). Interviews were audio taped and transcribed by a professional audio typist.

## Results and discussion

### Strengths and difficulties questionnaire

Individual inspection of the parent completed SDQs revealed that all children in the neuro-developmental disorder group scored ‘Abnormal’ on total difficulties and all subscales, with scores falling in the very high range (see Table 1 caption for bands). Individual inspection of parent scores for children in the neuro-typically developing group, showed that all scores were in the ‘Normal’ category for total difficulties and all subscales, with scores falling in the close to average range (Table 1). Individual inspection of the data sets revealed that no children in neuro-developmental disorder group and no children in the neuro-typically developing group should be considered to be moved into the other group. Children in the neuro-developmental disorder group scored significantly higher (representing greater difficulties) than the typically developing group on total difficulties (*F*(1, 32) = 73.96, *p* < 0.001) and the sub-scales: emotional problems (*F*(1, 32) = 41.19, *p* < 0.001), conduct problems (*F*(1, 32) = 15.77, *p* < 0.001), hyperactivity *F*(1, 32) = 28.61, *p* < 0.001) and peer problems *F*(1, 31) = 81.23, *p* < 0.001). Children in the neuro-developmental disorder group scored significantly lower (representing greater difficulties) than children in the typically developing group on the pro-social scale *F*(1, 32) = 20.68, *p* < 0.001) (see Table 1).

### Dog stress response checklist

In general parents reported infrequent observations of the specific behaviours listed, therefore as well as displaying data separately for each situation (Table 3), we also report frequency of observations across situations (Table 4). Dog biting was reportedly not observed by any parents in either group, whereas panting was the most frequently observed behaviour by parents in both groups. The dog cowering was reported to only be observed by parents in the NTD group. To assess whether there was a difference in observations of the dog’s behaviours by parents of NDD and NTD over the situations Fisher’s exact tests were conducted on the combined observations of behaviours over the eight situations. More parents of NTD children noted their dog rapidly running away from a situation (χ^2^ = 9.81, df = 1, *p* = 0.01), and more parents of NDD children noted their dog widening their eyes in a response to a situation (χ^2^ = 8.61, df = 1, *p* = 0.01). No other significant differences were observed.

**Table 3:** Proportion (%) of parents observing specific dog behaviours within eight situations with neuro-typically developing (n = 16) and children with a neuro-developmental disorder (n = 16).

	Resting		Playing		Walking		Dog eats		Child eats		Physical		Car		Running	
	NDD	NTD	NDD	NTD	NDD	NTD	NDD	NTD	NDD	NTD	NDD	NTD	NDD	NTD	NDD	NTD
Bite	0%	0%	0%	0%	0%	0%	0%	0%	0%	0%	0%	0%	0%	0%	0%	0%
Cower	0%	0%	0%	6%	0%	0%	0%	0%	0%	0%	0%	6%	0%	0%	0%	6%
Run away (fast)	0%	0%	6%	25%	0%	19%	0%	0%	0%	0%	0%	0%	0%	0%	0%	31%
Snap	6%	0%	6%	0%	0%	0%	0%	6%	0%	0%	6%	0%	0%	0%	0%	0%
Hide	0%	0%	6%	13%	0%	0%	0%	0%	0%	6%	6%	0%	0%	0%	6%	13%
Pace	6%	0%	6%	0%	6%	0%	6%	0%	0%	6%	0%	0%	0%	0%	0%	0%
Tense mouth	6%	0%	6%	6%	0%	6%	0%	0%	0%	0%	13%	13%	0%	0%	0%	6%
Run away (slow)	0%	0%	6%	25%	13%	13%	0%	0%	0%	0%	0%	0%	0%	0%	6%	19%
Growl	6%	0%	6%	0%	0%	6%	6%	6%	0%	0%	6%	6%	0%	0%	6%	6%
Shake	6%	0%	6%	13%	0%	6%	0%	0%	13%	0%	6%	0%	13%	19%	13%	6%
Walk away	6%	6%	25%	25%	0%	13%	0%	6%	6%	6%	19%	25%	0%	0%	0%	6%
Repetitive behaviour	19%	25%	13%	6%	6%	0%	0%	6%	0%	6%	6%	19%	6%	0%	13%	6%
Whine	6%	0%	19%	0%	13%	0%	6%	0%	6%	0%	0%	6%	19%	19%	0%	0%
Bark	6%	0%	19%	19%	0%	6%	13%	0%	13%	0%	6%	6%	6%	6%	19%	31%
Blink	6%	38%	6%	19%	6%	19%	13%	6%	6%	13%	25%	38%	19%	6%	0%	19%
Nose lick	19%	25%	6%	13%	6%	0%	6%	13%	6%	31%	31%	19%	6%	6%	6%	6%
Turn away	25%	25%	13%	13%	6%	0%	6%	13%	13%	19%	25%	25%	0%	6%	0%	13%
Lip lick	6%	6%	13%	13%	0%	6%	19%	25%	38%	50%	13%	25%	0%	13%	0%	13%
Wide eyes	19%	0%	19%	6%	6%	0%	13%	0%	13%	0%	6%	6%	6%	0%	13%	6%
Gaze Aversion	19%	0%	13%	0%	6%	0%	6%	13%	25%	0%	13%	25%	6%	0%	6%	6%
Stare	19%	44%	25%	13%	0%	6%	6%	0%	31%	56%	13%	31%	13%	6%	6%	0%
Pant	13%	6%	31%	31%	13%	44%	0%	6%	13%	13%	13%	19%	31%	31%	19%	50%

NTD: Neuro-typically developing child; NDD: Neuro-developmental disorder child. ***Resting***: Sitting, laying, sleeping with my child. ***Playing***: Playing with my child. ***Walking***: Walking with my child on or off the lead. ***Dog eats***: Dog is eating near my child. ***Child eats***: Child is eating near my dog. ***Physical***: Being stroked, cuddled, groomed by my child. ***Car***: In the car with my child. ***Running***: Running or chasing with my child.

**Table 4:** Frequencies and proportion (%) of parents with neuro-typically developing (n = 16) or children with a neuro-developmental disorder (n = 16) observing specific potentially stress-related behaviours in dogs across eight types of activity involving a child (see previous table list of situations).

	NDD	NTD
Number of times behaviour observed across eight situations (% data in brackets)		
**Bite**	0 (0%)	0 (0%)
**Cower**	0 (0%)	2 (2%)
**Run away (fast)**	1 (1%)	12 (9%)
**Snap**	3 (2%)	1 (1%)
**Hide**	3 (2%)	5 (4%)
**Pace**	4 (3%)	1 (1%)
**Tense mouth**	4 (3%)	5 (4%)
**Run away (slow)**	4 (3%)	9 (7%)
**Growl**	5 (4%)	4 (3%)
**Shake**	9 (7%)	7 (6%)
**Walk away**	9 (7%)	14 (11%)
**Repetitive behaviour**	10 (8%)	11 (9%)
**Whine**	11 (9%)	4 (3%)
**Bark**	13 (10%)	11 (9%)
**Blink**	13 (10%)	25 (20%)
**Nose lick**	14 (11%)	18 (14%)
**Turn away**	14 (11%)	18 (14%)
**Lip lick**	14 (11%)	24 (19%)
**Wide eyes**	15 (12%)	3 (2%)
**Gaze Aversion**	15 (12%)	7 (6%)
**Stare**	18 (14%)	25 (20%)
**Pant**	21 (16%)	32 (25%)

NTD: Neuro-typically developing child; NDD: Child with neuro-developmental disorder

### Interview data

Five salient themes emerged from the interviews which encompassed key issues that impacted on dog quality of life. A thematic rather than content analysis was performed therefore frequency counts of themes are not presented. Reported quotes from the thematic analysis were representative of similar statements and only used where multiple informants provided a similar response. A summary of the themes is presented in Table 5, with a brief commentary on each in the following text, before a more general discussion of our findings.

**Table 5:** Summary of main themes and sub-themes developed from the thematic analysis.

Main Themes	Sub-Themes
**Background Information**	Intensity of home environment Routine Dogs general interactions with people Dogs general behaviour Dogs health
**Interactions and Activities**	Child and dog interactions -Close contact -Low contact -Dog initiated Dog and child activities
**Threats and Uncertainties**	Meltdowns and tantrums Perceived threats by the dog
**Key Resources for Stress Management**	Safe haven Parent support Child education on dog interactions
**Child benefits**	(no sub-themes)

### Theme 1 –Background and daily management

#### Intensity of home environment

Many parents with NDD and NTD children commented on how busy and noisy their home was, with quiet periods noted when the children were at school, or in bed.

“Yeah, very loud, very lively. It’s never a break from it. It’s very ongoing, very loud and stressful!” (NTD SP)

“So literally, at seven o’clock, you can guarantee that the house is quiet. That’s it. And I guess for the dog in respect to that, he knows that that’s his time with us. Which is quite nice, he’ll come and well he’ll follow us until we actually sit down” (NDD SC)

One of the most troubling stress related conditions of dogs encountered by veterinarians is noise aversion (e.g. fearful response to environmental noise) [50–51]; highlighting a key stress factor for dog’s living in families with children. Given that treatments for general noise sensitivity are of limited effectiveness [52] it is important that families are aware of the need to provide a quiet space for the dog during peak time of busyness.

#### Routine

It was clear that having children imposed a routine within the household; both groups of children followed a fairly set pattern of activities during the day which meant the dog’s routine was stabilised around this. The importance of maintaining the set routine was particularly evident for households with children affected by neuro-developmental disorders.

“No, it’s quite a set routine. I suppose because we have to have such a–a rigid routine in the house with the boys, I think we’ve just transferred it onto the dogs.” (NDD DH)

Weekends and school holidays disrupted the dog’s usual routine, but many parents did not notice changes to their dog’s behaviour if their routine was altered, with the exception of missing a walk. Perhaps most importantly school holidays prevented the predictability of quiet times for the dog.

“If it’s a school holiday, then that affects his routine, because I’m not getting up and going out early with him, so he’ll maybe have his walk at sort of half nine, ten o’clock instead of going out at six o’clock in the morning. Weekends we tend to be a lot more relaxed, we don’t really have a routine too much at the weekends.” (NDD SA)

Stable routines are thought to be an important factor in determining a dog’s stress levels [4]; therefore, the routine that children impose on a household, and the importance of maintaining that routine, particularly for children with neuro-developmental disorders [53], may help protect the dog’s quality of life.

#### Dogs’ general interactions with people

Often the mother was viewed as the dog’s favourite person; this was typically the person who completed the interview. The number of people that the dog interacted with on an average day was fairly consistent, and comprised of immediate family members. Although households with NDD children made more references to home tutor visits, these were infrequent and generally very few parents of NDD and NTD children had regular help within the home. When visitors did arrive, many parents noted that this excited their dog, particularly child visitors, with comments of increased barking, agitation and proximity seeking to “mum”.

#### Dogs’ general behaviour

A number of parents from both groups viewed their dog as being ‘protective’, either over the family, or over their bed. Very few dogs were described as being ‘timid’ (e.g. shy, withdrawn). The majority of parents with both NDD and NTD children commented on how laid back and calm their dog was; there were many references to the dog enjoying being around people, particularly children, and that their dog was loving, sweet and even tempered.

“She has an amazing temperament and personality. She’s a very calm dog….she’s a really nice, calm, sweet dog, really gentle natured.” (NDD KA)

“She’s very laid back and relaxed.” (NTD DF)

Some parents with a child with a NDD observed that their dog was more quiet when around their child with a disorder, in comparison to other typically developing children in the household. This could reflect a number of factors, including that the dog had learnt to be quieter around the child to prevent negative interactions, or that the dog’s nature was to be more settled around a potentially more vulnerable member of the family. On the other hand, comments on the general excitability of the dog were more evident from parents with NDD children than parents with NTD children.

“He is bouncy, excitable, mischievous, naughty,” (NDD HB)

This hyper-responsiveness could be a sign of repeated activation of the sympathetic adrenal medullary system in emotionally stimulating events [54] and may suggest that the dog is experiencing frequent heightened emotions (positive or negative). It is also possible that it is more difficult for parents of NDD children to deal with hyper-responsive behaviours, due to increased parenting demands, therefore these parents are more alert to detecting and remembering these types of behaviours.

The dog’s obedience was typically referred to in relation to the child, with both groups of parents saying the dog would not always listen when the child gave the dog a verbal or physical command. It is possible that this reflects the lack of clarity in which the command is issued, which could be a source of confusion for the dog.

#### Dogs’ health (parent opinion)

Parents typically commented that their dog was in good health. Nonetheless, problems relating to the dog’s digestive system (sensitive stomach, inflammatory bowel disease and urine infections) were the most commonly reported problems, and this was particularly evident for dogs living with children with a NDD. Problems relating to skin conditions were also made more frequently by parents with NDD children. Eye and ear infections were also reported by both groups of parents. This is consistent with a hypothesis that reports minor ailments of these organ systems are possibly related to stress [4].

### Theme 2 –Interactions and activities

#### Child and dog interactions

Many parents with NDD and NTD children, commented that their dog seemed happy to greet and be with, their child and that they did not have concerns about leaving their child and dog alone together. Only parents with children with a NDD said they would not leave their dog and child alone together, but these sentiments were rare and acted as a preventive measure rather than based on past experience.

*Close contact*. A number of the child-dog interactions discussed by parents could be viewed as close contact. Comments centred around cuddling and kissing the dog were common from both groups of parents; both groups noted that sometimes the child could cuddle the dog too tightly for comfort.

“Part of his condition means that he’ll push too hard or he’ll stroke too hard, he doesn’t understand the depth of his, I don’t know how to describe it, but do you know what I mean, he pushes down too hard on him or if he puts his hands around (dog’s) neck he’ll be doing it as an embrace, as a cuddle, but I think sometimes he forgets that (dog’s name) is a dog.” (NDD–SP)

“If he’s given her a hug and it’s little bit tighter than she might like, she will just move, she’ll just get down and go somewhere else, and get out of his way.” (NTD–DF)

However, parents typically believed their dog liked being cuddled by their child, but did not actually know why they thought their dog liked it.

“Cuddling, like lots of cuddling when he kind of puts his arms and his legs round her and stuff like that, she likes that….. Because she just -, her ears are back up, she’s quite calm, I don't know, that’s a good question actually!” (NDD—KA)

Such behaviours (cuddling and kissing) have been related to dog-bite statistics [55] and suggest that parents need to improve their understanding and supervision of child-dog interactions [56] to promote child and dog welfare.

*Low Contact*. Parents, in both groups, commented on their child talking to, stroking, or giving tummy rubs to their dog, as well as a number of remarks on their dog enjoying sitting with their child watching TV. These interactions were viewed as being enjoyable by both the child and the dog and were perceived as being preferred over those of close contact by the dog.

“(Dog) doesn’t actually like being kissed or cuddled actually he just wants you to stroke him and he responds a lot better when (Child) just pats his head and tickles his ears and then he’s ok.” (NDD -CH)

“(Child) kind of strokes her ears and then (Dog) lays her head on her chest and moves her head a bit closer.” (NTD–AE)

Some parents with NDD children noted that due to their child’s condition the child did not spend much time sitting with the dog, due to hyperactivity or sensory sensitivities. This aspect of an NDD child’s disorder may have a positive impact on dog welfare.

“(Child) doesn’t pet him as in sit stroking him or anything, because (Child) doesn’t communicate in that way. But the dog will curl up next to him on the sofa or sit by his feet or if I’m sitting on the floor watching the TV, the dog will be at his side.” (NDD–TW)

*Dog initiated*. Parents of NDD and NTD children made references to their dog licking their child, particularly to the dog licking the child’s tears. The licks were sometimes referred to as the dog kissing the child. Some parents also referred to their dog initiating a hug.

“He does like to sit on (Child’s) shoulder, which I know sounds a bit weird for a dog, but I've got quite high backed sofas and (Dog) will walk along the back of the sofa and sort of stand with his front two paws on (Child’s) shoulder and give him a massive kiss.” (NDD—KH)

“She’ll also sit next to my little boy and lick him”. (NTD–DF)

It is possible that the hugging type behaviour shown by the dog is a learnt technique to release the embrace, particularly with NDD children, as there was some suggestion that parents rewarded the dog for ‘hugging’ the child which would result in release.

“What (Dog) won’t do is pull out of the embrace, which is bizarre because he does with everybody else, but with (Child) if (Child) puts both his arms around his neck to sort of give him a hug, (Dog) sometimes puts his chin on his shoulder and it looks like he’s cuddling him, but I still believe that he probably doesn’t like it if I’m honest. But he sticks his chin on (Child) shoulder and then if I see that happening it’s almost like (Dog) saying ‘is that what me to do?’ and at that point I will say ‘well done (Dog) and I’ll take something out of my pocket and give him and then I will sort of break them up from that point.” (NDD–SP)

Indeed, it has been suggested that although a dog’s response when their owner cries may look like empathy it is more likely to reflect a learned association between behaviour and reward [57]. It is therefore important that parents are conscious not to anthropomorphise their dog’s behaviours as this may lead to misinterpretation of cues that signify the dog is unhappy with the situation.

Parents from both groups also reported behaviours from the dog which may represent a strong attachment bond between the child and the dog, such as the dog looking lovingly at their child and the dog moving closer and leaning into their child [58]. There were a few comments that the dog would favour sitting with the children when they were eating than the adults in the hope of receiving a food-treat.

#### Child and dog activities

Parents of NDD and NTD children made reference to similar shared activities that the dog and the child enjoyed together; these were typically high energy activities such as playing ball, chase, and doing obstacle courses together. Parents also talked about how their dog liked to join in with reading.

“She (Dog) likes to sit with us when we read so she always tries to get in the middle. If we’re doing reading for school she loves that, she wants to sit in the middle of me and my other child while we read the book.” (NDD -LF)

“We have quiet time before bed reading a book and things she would like get in-between the two of them or with whoever’s reading the book.” (NTD -AC)

This could be because it is a chance to sit quietly with the family, but may have important positive impacts on the child’s development [59]. Both NDD and NTD children had been observed playing dress-up with their dog, which parents believed their dog was willing to participate in. Dog walking with the children was largely viewed as being enjoyed by the dogs. One parent in the NDD group noted that their dog preferred walking with their child because the walk was conducted at a steadier pace.

“(Child’s name) doesn’t go too fast and he’ll, sort of, he’ll stop and interact a little bit with her and say ‘come on you’, encouraging her to come on.” (NDD—DH)

However, both group of parents commented that the child does pull the dog around on the lead.

“Everybody always want to walk him so (Dog) gets sort of dragged about and he’s like this isn’t really walking, like a dog being hoyked around by various children. (NTD—LD)

“(Child) sometimes gets his collar and tries to walk round with him and sometimes (Dog) is like ‘I don’t want to go, I want to stay.” (NDD–AB)

### Theme 3—Threats and uncertainties

#### Meltdowns and tantrums

Meltdowns (i.e. reaction to being overwhelmed) and tantrums (i.e. angry or frustrated outburst) were noted by both groups of parents as being a regular occurrence, but more frequent for children with a NDD, occurring multiple times a day. Parents typically believed that these events caused some form of stress to the dog. The dog was viewed as being stressed when it showed overt behaviours, such as barking, jumping up and shaking.

“You would see her (Dog) hiding, possibly shaking a little bit, her eyes would get quite wide. She’s obviously got wide eyes anyway because she’s a King Charles. But you can see there’s a bit of a–not horror, but not liking the situation.” (NDD -DH)

“The dog will tend to either retreat into his bed, his little corner area where he sits, and you know, you can see him occasionally sort of hankering down and trying not to make eye contact with (Child).” (NDD -ST)

“She (dog) kind of gets a bit agitated when one of them is upset or anything.” (NTD–EE)

However, a small number of parents from both groups stated their dog showed no reaction to meltdowns.

“She had a big one, a big meltdown, this morning and the dog just carried on, didn’t even sort of look really. (NTD -EH)

“It doesn’t seem to distress him at all.” (NDD–JM)

Some parents to NDD and NTD children noted that their dogs would seek closer proximity to their child during a meltdown. Behaviours such as scratching at the child and laying close to the child were mentioned. Some dogs, living with an NDD child, were regularly observed to spontaneously lay on the child during a meltdown.

“When he starts kicking off, the dog like scratches with one of her paws on his leg and he just cuddles up to her and he’s still crying but he just cuddles up to her and normally covers them both over with a blanket and then he’s fine after half an hour or so.” (NDD–AB)

“She’ll go over and investigate and to be honest with (Child) she’ll probably go and sit with her.” (NTD–LT)

“(Dog) will just go and place her weight on him, either laying across him or she’ll be on him physically somehow” (NDD–VH)

Whilst behaviours such as laying on the child may be trained by autism assistance dogs to provide deep pressure therapy to help soothe the child [40], it is interesting to note that this may be a natural spontaneous behavioural response for some dogs in some circumstances. These behaviours may reflect a tendency to provide comfort to the child, or they may be an expression of attention seeking or arousal which is misunderstood by the parent.

“He’ll crouch down, he will roll over, he does quite a lot to try and calm him down. Generally speaking, which I find quite interesting is he can bring (Child) out of a meltdown.” (NDD–CB).

Only one parent (NDD) had attempted to prepare their dog for meltdowns and tantrums by controlling exposure to tape-recorded screams and offering rewards. This parent believed their dog coped well with tantrums and meltdowns. Given that continued exposure to noise rarely results in habituation [60], this type of conditioning work, associating meltdowns with a positive emotional response, may be an effective strategy.

Parents also perceived that their dog did not like shouting anyone (parent of child) shouting in the house. Therefore, whilst meltdowns and tantrums may be more frequent and perhaps extreme with children, shouting and volatile behaviour is not a unique welfare risk to dogs living with children.

#### Perceived threats by the dog

Parents of NDD children made a number of remarks on their child hitting the dog due to their problems controlling their impulses.

“Sometimes (Child) can be quite horrible to the dog. You know, he will lash out at the dog or something. He shouts at him more. I mean he used to hit the dog, but he doesn’t do that, very rarely now.” (NDD -SR)

One parent, in the NDD group had trained their dog to prevent their child from hurting themselves by training the dog to block with their body, so when the child directed hits at their head they made contact with the dog instead. The parent noted that the child would stop this behaviour once they realised it was the dog being hit, not themselves.

Comments on the dog being physically hurt were, however, not unique to parents of NDD children, with parents of NTD children commenting on their child jumping on the dog, or laying over the dog.

“We were in our campervan a couple of weeks ago, and he (Child) was chasing after her (Dog), and I was busy doing something so I hadn’t entirely see what was going on and that she was so worried. But she ended up sort of coming in, was quite worried and shaking, tried to hide.” (NTD—JN)

“(Child) will kind of sometimes just flop onto her (Dog), ha ha!” (NTD–KA)

A number of games were mentioned by both groups of parents that the dog did not enjoy playing with the child, or did not like it when the child played these games near them. These included references to ball games, chase, rolling on the floor, skateboards and the child bouncing. These games appear to be similar in that they impose a certain degree of risk to the dog of being harmed, such as being run over by wheels, or being bounced on or stood upon. Although rare, parents in both groups noted that their child had, at times, ran the dog over with wheeled toys, dragged the dog and pulled the dogs tail.

“He doesn’t like (Child) skateboard or his scooter if (Child) gets them out he’s gone like a shot.” (NTD–CT)

“He puts up with a lot of tail pulling and poking since he was little and he’s only snapped at him once which I think is pretty good considering what he’s put up with, a bit grumpy but not particularly nasty I suppose.” (NDD–MB)

It should be noted that some of these games were also mentioned by some parents as being enjoyable shared activities, and may highlight individual differences in a dog’s preferred activities, and/or suggest individual variation in parent’s interpretation of the dog’s behaviours during enjoyable and unenjoyable activities.

Comments were made from both groups of parents about the dog not enjoying being groomed or bathed by the child. Only parents with NDD children also reported that their dog did not like being in the car with their child. It is possible that this is because when the child is in the car the dog is in a confined space with loud noises, but also that the dog is hot. Indeed, one parent, in the NDD group, remarked that they do not open the window for the dog when the child is in the car too, and dogs may rapidly overheat in warm cars.

### Theme 4—Key resources for stress management

#### Safe haven

One of the most frequently mentioned reaction was for the dog to remove themselves from an unpleasant situation, by either seeking a place to hide (in or out of the room), or by taking themselves off to their safe haven. Specifically, this behaviour was regularly mentioned in relation to meltdowns and tantrums. Parents noted that their dog gave a ‘sigh of relief’ when they took themselves off and often encouraged the dog to go to their bed during potentially anxiety provoking situations.

“She (Dog) loves her crate because that is her space and she knows that and the children don’t go in the crate. So yeah, she will, she’ll just take herself off in there and she’ll lay in her bed and she’ll just curl up and you can hear her, you know, and you’re just like yeah, that’s her sigh of relief.” (NDD—VH)

“He sighs and goes to lie on his bed”. (NTD–AM)

Many parents mentioned that the dog would be happy to come back into the room with the child once the situation had calmed down, and would often seek the child upon return. This suggests that allowing the dog to escape from the situation is effective in allowing the dog to cope and in protecting the dog’s relationship with the child.

(After the meltdown)…. *“He’ll (dog) come back out and he’ll go and sit next to (Child) sort of ready for a cuddle or something*.*” (NTD–CT)*

“Once (Child) has calmed down and normally he’ll (Child) go up to bed and he’ll go under his quilt and lay in bed right under his quilt and that’s when (Dog) will then go and find him.” (NDD–KH)

A number of dogs in both groups had more than one potential safe haven areas to choose from, one which was their spot in the room (e.g. a blanket on the sofa) and one which was their designated bed. Some dogs did not have their own bed and would choose to hide, or find a quiet room to sit in. Allowing the child near the dog’s bed was generally discouraged, but this did occur in some instances from both groups of children. Although rare, there were also reports of dogs being protective over their bed, growling if the child approached.

#### Parent support

Parents appeared to be a strong source of support to dogs living with NDD and NTD children. Parents noted that the dog would often look to them for support. Dogs were observed to look over with large eyes to parents and physically go find the parent. Child initiated situations that would prompt the dog to seek the parent included meltdowns and tantrums and possibly threatening interactions for the dog, such as being run over by toys. Parents commented that they would reassure the dog, through touch and voice in these circumstances, which is consistent with the caring style within secure attachments [58].

“She’ll sort of look at me and I’ll rub her ears and talk to her and stroke her. Sometimes it can be for a few seconds and then she’ll go off as though she just needs that little bit of touching base and then she’ll be off again.” (NTD–EH)

“Yeah like if he was playing with her in the living room and decided that he wanted to know where I was and I was in the Kitchen he would come and find me, he would equally do that if he thought he didn’t want to play anymore.” (NDD–HB).

Dogs, from both groups of families, would also show signs of stress if they could not access the parent, looking around the house for them and showing separation anxiety. Research suggests that owners can act as a secure base for dogs during exposure to stressful stimuli, and is associated with a reduction in heart rate responses [61].

For parents to provide the necessary support to their dog it is important that they understand their dog’s expressions of emotion. Parents noted that their dog was happy from their eyes, their relaxed body posture and the position and mobility of the tail. Unhappiness was indicated by vocal noises (barking and whining), stiffening of the body, facial expressions (lip licks, gaze aversion, ears back, yawning and panting), pacing and disinterest in food. Although rare, there were a few references from both groups of parents were made to their dog growling or snapping at their child if they do not welcome an interaction. Parents from both groups commented that they found it hard to think of, or overtly notice, specific behaviours their dogs did when they were feeling anxious. It should be noted that parents completed the dog stress checklist prior to taking part in the interview, as such the behaviours listed in the checklist may have prompted the parent to recognising these behaviours.

#### Child education on dog interactions

Parents of both groups of children encouraged their child to praise their dog, and take part in dog training activities. References to teaching the child about the dog’s behaviours were made frequently by parents to a child with an NDD. This may be because these parents believe that their child represents a greater risk to their dog, and therefore are more attentive to preventing negative interactions. Parents with NDD children commented that their child had difficulty understanding the dog, it was challenging to teach their child about the dog’s behaviours, and instead it was easier to teach the dog how to respond.

“It’s hard for (child) to explain to him not to do something. So we have explained that he doesn’t always stop which is why we taught (dog) to move out to the situation rather than, it’s easier to teach (dog) to move than it was to teach (child) to stop.” (NDD—CB)

### Theme 5: Child benefits

When asked about the relationship between their dog and child parents overwhelmingly responded with a range of superlatives. Parents to NDD and NTD children believed that their child loved their dog and that their dog gave their child responsibilities. In particular parents with children with a NDD commented on the importance of the dog’s friendship to their child, the emotional support provided by the dog and role the dog played in helping their child communicate.

“She’s got a speech delay as part of her diagnosis and she is coming on nicely with that now a lot of her speech is modelled on him (the dog) he was the first person she ever verbally asked to play which was incredible….I guess at the moment she’s obviously an only child but I guess in a similar way to what she possibly would have done with a sibling.” (NDD—HB)

“I kind of used him to teach her manners as well, so for example if he’s lying in the middle of the room and she wants him to move she will say (Dog) can you move please and when he moves thank you and everything so it’s been good from that respect, but she’s using her manners and learning that through him.” (NTD—NW)

However, although rarely, parents with a child with a NDD believed that sometimes their child was jealous of the dog and the attention the dog received. Parents also stated that their dog would cue the parent to check on their child, or recognise that their child was notwell. One parent, with a child with a NDD, reported an event where their child had locked themselves in their bedroom with suicidal intentions and the dog had raised the alarm by barking and scratching at the bedroom door. These observations are consistent with research highlighting the important role that pet dogs can play in healthy child development [62–64].

## General discussion

This is the first research that explores quality of life for dogs living in different types of families. It should be noted that this exploratory study did not aim to conduct a case-matched control comparison between the two family groups; instead by gathering the perspectives of families with neuro-typically and neuro-atypically developing children we report perceptions and beliefs about issues which may quality of life for pet dogs living in these homes. It should also be pointed out from the start that the discussion of the results should be taken in context. As with most qualitative studies the sample size is relatively small, with a larger proportion of dogs living with male compared to female children, particularly in the neuro-developmental disorder group. Additionally, the large majority of respondents were female (i.e. the mother). Furthermore, whilst the researchers strived to synthesise the data as it emerged, without imposing their beliefs and expectations, it is likely that our backgrounds in psychology and animal behaviour have to some degree influenced our interpretations.

It is difficult to infer whether observations of dog behaviours reflect specific responses displayed by dogs living with NDD versus NTD children, or whether they reflect the parent’s ability to identify their dog’s reactions. Indeed, a considerable number of parents in the NDD group (25%) had attended assistance pet dog workshops, which often train parents to recognise more subtle cues of stress in dogs. This may well explain why more parents in the NDD group reported their dog ‘widening their eyes’ in response to a child-dog interaction than parents in the NTD group. We highlight that the small sample size of this study means quantitative reports should be interpreted as pilot data only, rather than substantive claims of evidence. Therefore, the primary focus of this discussion is on considering the implications of parent’s perceptions of dog quality of life based on the qualitative data collected. Nonetheless, the quantitative data reported here indicate the importance of allowing the dog an escape route to easily and quickly remove themselves from an interaction they do not feel comfortable with and the need for parents to be aware of subtle as well as overt behavioural responses [17–18].

The qualitative data highlights that children bring several positives to dogs’ lives. In particular, having children imposed a routine to the house, and the importance of maintaining a stable routine was heightened in families with a child with a neuro-developmental disorder. Children, from both groups, also facilitated high-energy activities (such as playing ball) and there was typically a strong bond reported between the child and dog. These observations are in contrast to previous research exploring quality of life of service dogs to children with ASD [40], which reported reduced access to recreational activities and routine within the home. It is possible that this difference reflects the unique role played by a trained service dog in comparison to an untrained family dog, whereby the former is on constant ‘work mode’.

A number of negative factors were evident for dogs living with children in general, including: the risk of meltdowns and tantrums, invasion of personal space caused by tight embraces, kisses and being in the car with the child, over stimulation caused by child visitors to the house, rough play and toys with wheels, and child physical aggression. This is comparable with the findings reported by Burrows et al. [40] who identified risks to dog quality of life around potentially negative attention from a child with autism (e.g. rough play / contact), but it seems the risk extends to families with neurotypically developing children too. In general, the potentially negative factors observed here were evident in both groups, with the exception of stress at being in the car with the child, which was only mentioned in families with a child with a neuro-developmental disorder. However, the frequency, intensity and duration of meltdowns and tantrums for children with neuro-developmental disorders was increased, indicating dogs in these families may have greater repeated exposure to this stressor, which may have clinical relevance. Potentially as a result of this, some families in both groups reported stress-related health conditions in their dog, such as skin and gastro-intestinal issues, this was noted particularly by families with children with a neuro-developmental disorder. However, we did not provide clinical assessment to corroborate these claims.

It is clear from this data that there are commonalities between parents’ beliefs on what affects dog welfare Predictability and routine is an important issue encompassing the frequency, intensity and duration of events and experiences such as child visitors, meltdowns and tantrums, noise levels (quiet times) and being in the car with the child. Child and dog physical contact is another key issue, relating to the child’s sensory preferences and understanding, incorporating events such cuddling, kissing, striking out, grooming and bathing the dog. Child and dog play is a further key issue, including the importance of enjoyable shared high-energy activities and the perceived threat from certain toys (e.g. wheeled toys) and games (e.g. bouncing).

Having identified the issues which potentially affect quality of life for dogs living within different family groups we highlight the need for the development of a stress audit for pet dogs. Indeed, it has recently been identified that there is a clear need for the development of validated tools to assess quality of life in pet dogs [65]. The development of such an audit may include assessing the frequency of the issues reported here (e.g. meltdowns and tantrums), and their consequences on the physical and emotional well-being of the dog. To provide a comprehensive assessment of dog quality of life the proposed audit should be used in conjunction with expert clinical consultation. The implementation of this audit has the potential to act as a monitor for early intervention to prevent catastrophic events such as dog bites and animal neglect due to dissatisfaction with the relationship, as well as help to maximise the potential benefits of the human animal bond. We also identified important protective factors for dog welfare: having a safe haven to escape to, parent’s awareness of stress signs and child education in dog-interaction (typically parent-led). As such, this research draws attention to the importance of parent training to educate on key risks and coping strategies.

Our research is strengthened by the national recruitment campaign, which included respondents from across England. However, it may be that families that had particularly positive experiences of dog-ownership, or were particularly sensitive to their dog’s wellbeing were motivated to participate in the study. Given the qualitative nature of this study, it is challenging to corroborate the findings. Future research could video record the dog and family interactions, as well as combining parent report data with physiological measures and clinical assessments to provide a more objective assessment of pet dog quality of life [65]. Additionally, although this qualitative study has allowed us to identify key issues to consider in relation to pet dog welfare we do not make quantitative comparisons between family groups at this stage; the development of the proposed audit would enable quantitative comparison of risk from living with children with different neuro-developmental statuses, but may also allow monitoring of the dog and so serve as a useful preventive or management tool. Moreover, a quantitative comparison may enable comparisons of the effectiveness of child, family or dog focussed interventions which promote dog wellbeing. For instance, in this study we did not record whether the child was taking medication or engaged in any cognitive-behavioural therapy interventions, which may affect the nature of their interactions with the dog. Furthermore, large scale quantitative studies could include consideration of individual differences in the child (e.g. severity of condition, gender) the dog (e.g. breed, weight, age) and the parent (e.g. gender, attachment) in determining dog quality of life in family homes.

The findings of this exploratory study provide a valuable first insight into pet dog’s quality of life in children with and without neuro-developmental disorders. The differences between the two groups may not be as unique as might be thought with many commonalities in what was reported between the families with children who are neuro-typically developing and those with a neuro-developmental disorder. This study brings valuable information on the potential aspects that could be included in a future instrument for screening the impact of child-dog interactions of dog quality of life, in combination with veterinary medical assessment. We also highlight the bidirectional benefit for pet dogs and children and the need for future research in this area.
